# Iodide of Potassium in Frontal Headache

**Published:** 1882-04

**Authors:** 


### IODIDE OF POTASSIUM IN FRONTAL HEADACHE.

Dr. Haley states, in the Australian *Medical Journal* for August, that for some years past he has found minimum doses of iodide of potassium of great service in frontal headache. A heavy dull headache situated over the brow, and accompanied by languor, chilliness, and a feeling of general discomfort, with distaste for food, which sometimes approaches to nausea, can be completely removed by a two-grain dose dissolved in half a wineglass of water, and this quietly sipped, the whole quantity being taken in about ten minutes. In many cases the effect of these small doses has been simply wonderful. A person who a quarter of an hour before was feeling most miserable, and refused all food, wishing only for quietness, would now take a good meal and resume his wonted cheerfulness. The rapidity with which the iodide acts in these cases constitutes its great advantage.

